# *In vitro* Anti-mycobacterial activity of selected medicinal plants against *Mycobacterium tuberculosis* and *Mycobacterium bovis* Strains

**DOI:** 10.1186/1472-6882-13-291

**Published:** 2013-10-29

**Authors:** Abdella Gemechu, Mirutse Giday, Adane Worku, Gobena Ameni

**Affiliations:** 1 Department of Medical Laboratory Sciences, College of Health and Medical Science, Haramaya University, Harar, Ethiopia; 2 Aklilu Lemma Institute of Pathobiology, Addis Ababa University, Addis Ababa, Ethiopia

**Keywords:** Antimycobacterial activity, Medicinal plants, MIC, REMA, M. tuberculosis & M. bovis strains, Ethiopia

## Abstract

**Background:**

Tuberculosis (TB) is a global burden with one –third of the world’s population infected with the pathogen *Mycobacterium tuberculosis* complex and annually 1.4 million deaths occur due to the disease. This high incidence of infection and the increased rate of multi-drug resistant and extensively-drug resistant strains of the organism further complicated the problem of TB control and have called for an urgent need to develop new anti-TB drugs from plants. In this study, the in vitro activity of root of *Calpurnia aurea*, seeds of *Ocimum basilicum*, leaves of *Artemisia abyssinica*, *Croton macrostachyus*, and *Eucalyptus camaldulensis* were evaluated against *M. tuberculosis* and *M. bovis* strains.

**Methods:**

Five Ethiopian medicinal plants, root of *Calpurnia aurea,* seeds of *Ocimum basilicum,* leaves of *Artemisia abyssinica, Croton macrostachyus,* and *Eucalyptus camaldulensis* used locally for the management of TB. They were investigated for in *vitro* antimycobacterial activity against *M. tuberculosis* and *M. bovis* strains. 80% methanolic extracts of the plant materials were obtained by maceration. The antimycobacterial activity was determined using 96 wells of microplate with the help of visual Resazurin Microtiter Assay.

**Results:**

The crude 80% methanolic extracts of the root of *C. aurea,* seeds of *O. basilicum,* and leaves of *A. abyssinica, C. macrostachyus,* and *E. camaldulensis* had anti-mycobacterial activity with minimum inhibitory concentration (MIC) ranging from 6.25–100 μg/mL. The MIC of 80% methanol extracts in the order mentioned above ranged 25-100 μg/ml and 12.5-75 μg/mL, 25–100 μg/mL and 25–50 μg/mL, 6.25-50 μg/mL and 12.5-50 μg/mL, 12.5-100 μg/mL and 18.25-50 μg/mL and 6.25-50 μg/mL and 12.5-50 μg/mL, respectively for *M. tuberculosis* and *M. bovis* strains.

**Conclusions:**

The results support the local use of these plants in the treatment of TB and it is suggested that these plants may have therapeutic value in the treatment of TB. However, further investigations are needed on isolating chemical constituents responsible for eliciting the observed activity in these plants.

## Background

Tuberculosis (TB) is caused by a set of closely related mycobacterial strains such as *Mycobacterium tuberculosis, M. bovis, M. africanum* and others, known collectively as the *M. tuberculosis* complex (MTC) [1]. Currently about one –third of the world’s population is assumed to be infected with the tubercle bacillus [2,3]. It is responsible for 1.4 million deaths per a year [4]. Moreover, up to 50 million people are said to be infected with drug-resistant forms of TB from which about 500,000 cases of multi-drug resistant (MDR) TB worldwide per a year [5].

Management of TB/MDR-TB patient requires intense multi-chemotherapy for at least six months to two years. It is very hurtful to a patient’s health due to high levels of drug toxicity and its adverse effects [6-8]. The emergence of MDR TB and extensively-drug resistant (XDR) TB to the medicines now in use makes urgent search for new anti-TB agents worldwide [9,10]. Medicinal plants offer a great hope to overcome these needs because of their chemical diversity and their significant role in the drug sighting and development [11]. They are also recognized as a useful source of highly active antimycobacterial metabolites [12].

In Ethiopia there are many plant species reported to be used traditionally to treat TB and respiratory tract infections. For instances, leaves and fruits of *Allium ursinum* were locally used for the treatment of bronchitis and TB respectively in Central Zone of Tigray [13]. It was also reported that locally available plant species such as *Croton macrostachyus* and *Oenanthe procumbens* in Farta wereda of Amhara region [14] and *Arisaema schimperianum* around Bale mountains national park [15] are to used to treat TB respectively. Likewise, roots of *Calpurnia aurea* are used for lung TB [16]. Additionally it was reported that fruits/seeds of *Ocimum basilicum* and *Ocimum americanum,* leaves of *Eucalyptus camaldulensis, Artemisia afra* and *Artemisia abyssinica* are used for cough and TB [17]. Leaves of *Aloe pubescens*, root of *Indigofera amorphoides* and *Psydrax schimperiana* are reported to be some of the medicinal plants locally used to treat TB and other respiratory illness and other diseases [18]. Moreover, in Chifra District, Afar region, it was reported that *Kanahia laniflora* and *Aloe species* were locally used for treatment of TB [19]. However, their efficacy remains unknown. Thus, the aim of this study was to evaluate the *in vitro* anti-mycobacterial activity of root of *Calpurnia aurea,* seeds *Ocimum basilicum,* leaves of *Artemisia abyssinica, Croton macrostachyus,* and *Eucalyptus camaldulensis* against *M. tuberculosis* and *M. bovis* strains.

## Methods

### Study design

This study was conducted at Aklilu Lemma Institute of Pathobiology (ALIPB), Addis Ababa University (AAU) using an experimental study design. Five plant species, namely *C. aurea, O. basilicum, A. abyssinica, C. macro-stachyus,* and *E. camaldulensis* were selected for their *in vitro* activity test against *M. tuberculosis* and *M. bovis* strains*.* Susceptibility tests and MIC of the extracts were determined using Resazurin Microtiter Assay (REMA)*.* Rifampicin was used as positive control while drug and extract free medium were used as negative controls.

### Collection of plants materials

The leaves of *C. macrostachyus*, roots of *C. aurea*, and seeds of *O. basilicum* were collected from Addis Ababa. Leaves of *E. camaldulensis,* and *A. abyssinica* were collected from Harar (525 km East of Addis Ababa) and Muka Turi (Selale/North Shoa Zone) 80 km north of Addis Ababa, respectively (Table 1). The identities of each plant specimen were identified by Dr. Mirutse Giday (a botanist) at the Endod and Other Medicinal Plants Research Unit, ALIPB. A voucher specimen of each plant was deposited at the Endod and Other Medicinal Plants Research Unit, with voucher numbers AG-01 (*O. basilicum*), AG-02 (*C. macrostachyus*), AG-03 (*C. aurea*), AG-04 (*E. camaldulensis*), and AG- 05 (*A. abyssinica*). All parts of the plant materials were made to become dry in an open air and protected from direct exposure to sunlight. The dried plant materials were separately powdered to suitable size by using mortar and pestle, and then made ready for extraction.

**Table 1:** **Medicinal plants used for antimycobacterial activity against *Mycobacterium tuberculosis *** **and *Mycobacterium bovis *** **strains**

**Plant species**	**Family**	**Vernacular name**	**Parts used**	**Voucher specimen**	**Place of collection**
*Ocimum basilicum*	Lamiaceae	Basobila (Amharic (Amh))	Seeds	AG-01	AA (from market)
*Croton macrostachyus*	Euphorbiaceae	Bisana (Amh)	Leaves	AG-02	ALIPB compound
*Calpurnia aurea*	Leguminosae	Digitta (Amh)	Roots	AG-03	ALIPB compound
*Eucalyptus camaldulensis*	Myrtaceae	Key bahirzaf (Amh)	Leaves	AG-04	Harar
*Artemisia abyssinica*	Asteraceae	Chikugn (Amh)	Leaves	AG-05	Muka Turi

### Preparation of the extract

The powdered plant materials were weighed using analytical balance and prepared for solvent extraction. The crude 80% methanolic extracts of the plant materials were obtained by maceration. The total crude 80% methanolic extracts were obtained by soaking powdered plant material (about 50 g) in 400 mL methanol for 48–72 h on an orbital shaker [20]. Extracts were filtered using Whatman No.1 filter paper and concentrated using a rotary evaporator (Laborota 4000, SN 090816862, Germany) in a water bath set at 40°C. The dried methanolic extracts obtained from each plant were air-dried then packed in glass bottles with proper labeling for future reference. The extracts were kept refrigerated and far away from light. Stock solutions were prepared in dimethyl sulphoxide (DMSO) at a concentration of 20 mg/mL and stored at -20°C until use [20,21]. Finally, all the crude 80% methanolic extracts of each plant species were tested for antimycobacterial activity.

### Test organisms

*M. tuberculosis* strains including (H37Rv, SIT777, SIT73, SIT26, SIT37, SIT1688, SIT336, SIT149, SIT53, and SIT54) and *M. bovis* strains (SB1176, SB1953 and SB0133) were obtained from TB laboratory of ALIPB and were used for evaluation of plants crude extracts.

### Preparation of Inoculum

The isolates grown on Lowenstein Jensen medium (LJ) were sub-cultured in Middle Brook 7H9 broth supplemented with OADC at 37°C for 14 – 21 days. The anti-mycobacterial activities of the extracts were evaluated against ten *M. tuberculosis* strains and three *M. bovis* strains by maintaining on Middle Brook 7H9 medium for about 14–21 days. The bacterial suspension was homogenized by vortex shakeup and the turbidity was adjusted in agreement with tube which is the scales of McFarland no.1 (3.2 × 10^6^ cfu/mL). The inoculum was prepared diluting the bacterial suspension in the proportion of 1:20 in Middle Brook 7H9 broth medium. This diluted suspension (100 μL) was used to inoculate each well of the plate [22].

### Anti-mycobacterial activity tests

The antimycobacterial activity of 80% methanolic crude extracts of *O. basilicum* seeds*, C. aurea* root*, C. macrostachyus, A. abyssinica* and *E. camaldulensis* leaves were tested using the REMA. The susceptibility test was accomplished in 96 microplates (wells) using the resazurin as an indicator of cellular viability or growth inhibition. Working solutions of the tested extracts were diluted in Middle Brook 7H9 broth supplemented with OADC to obtain final sample concentrations that ranges from 0.78 μg/mL to 100 μg/mL. Rifampicin was dissolved in DMSO and used as positive control drug and extracts/drug free medium with strain suspensions were used as negative control. One hundred micro liters of Middle Brook 7H9 broth and the test inoculum were added to all testing wells, as well as to the drug/extract-free control wells. Then, one hundred micro liters working extract solutions were poured into the first well of each row from which two-fold dilution series were made through the micro plate column. Each extract concentration was assayed in duplicate. Each micro plate was then sealed with parafilm and incubated for 5–7 days at 37°C in normal atmosphere. After the incubation period, 25 μL of resazurin 0.02% w/v was added to each wells and re-incubated at 37°C for 24 h for color development. The visual MIC was defined as the lowest drug/extract concentration that prevented the color change of resazurin reagent from blue to pink. Blue color in the well was interpreted as there is no mycobacterial growth and pink color was scored as growth occurrence [23,24].

### Determination of the Minimum Inhibitory Concentration (MIC)

MIC was determined by using the REMA in 96 well micro titer plates. One hundred micro liters of Middle Brook 7H9 broth and *M. tuberculosis* and *M. bovis* strains were dispensed into all wells of a sterile 96-well microtitre plate. In the first column (no.1 well of all plate), one hundred micro liters of extracts were added to each first wells using a unique pipette for each extracts. The extracts were mixed thoroughly and fifty micro liters of extracts were transferred to well 2 to well 8 from which fifty micro liters were discarded. Well 9 up to well 12 were served as sterility and negative controls. The working solution of extracts (100 μg/mL) were diluted out across a 96-well in a two-fold serial dilution to give final testing concentrations of 50, 25, 12.5, 6.25, 3.12, 1.56 and 0.78 μg/mL. The same procedure was followed for rifampicin with the initial concentration of 32 μg/mL with subsequent dilution to the final testing concentrations 16, 8, 4, 2, 1, 0.5, 0.25, 0.125 and 0.06 μg/mL. The plates were then incubated for 5–7 days at 37°C. After 7^th^ day, 25 μL of resazurin was added to all wells and re-incubated overnight for development. The MIC was defined as the lowest concentration of the extracts/drugs that prevented a colour of resazurin to be changed from blue to pink (visual determination) [24-27]. According to Ramos [22], extracts were considered as active if they inhibited growth of mycobacterium at MIC ≤ 100 μg/mL. Each extract was tested in duplicate against each strain.

### Data analysis

The experimental results were computerized using EpiData version 3.1 and STATA version 11 statistical software for data entry and analysis purposes respectively. The MIC results were presented as mean value. The t-test was employed to test significance for the difference between 80% methanolic extract results of each plant and rifampicin against *M. tuberculosis* and *M. bovis* strains. All the data were analyzed at 95% confidence interval and considered as statistically significant.

### Ethical consideration

The study was conducted after its approval by the Institutional Review Board of ALIPB. The test was done in the TB laboratory where the necessary protective wears such as respirators and gloves as well as safety cabinets were used, to minimize the risk of exposure to *M. tuberculosis* and *M. bovis* isolates*.*

## Results and discussion

### Anti-mycobacterial activity of the crude extracts

Crude 80% methanolic extracts of all the five plants, namely leaves of *C. macrostachyus,* seeds of *O. basilicum,* root of *C. aurea,* leaves of *E. camaldulensis* and *A. abyssinica* were screened for their antimycobacterial activity against *M. tuberculosis* strains (H37Rv*,* SIT777, SIT73, SIT26, SIT37, SIT1688, SIT336 ,SIT149, SIT53, SIT54*)* and *M. bovis* strains (SB1176, SB1953 and SB0133) by using REMA had showed antimycobacterial activity against *M. tuberculosis* and *M. bovis* strains with the mean MIC values ranging from 6.25 to 100 μg/mL. Selective activity of five 80% methanolic crude extracts and rifampicin against all strains of *M. tuberculosis* and *M. bovis* were observed (Table 2).

**Table 2:** **Mean of MIC values of 80% methanolic crude extracts of five medicinal plants against ten *M. tuberculosis *** **strains and three *M. bovis *** **strains using visual REMA**

**Strains**	**MIC (μg/ml)**						
	**Strain name**						
		**Ob**	**Ca**	**Cm**	**Aa**	**Ec**	**Rif**
H37Rv	MTB	25	37.5	12.5	6.25	25	2
SIT777	MTB	50	50	100	50	25	4
SIT73	MTB	50	25	25	25	6.25	0.25
SIT26	MTB	75	50	25	50	50	2
SIT37	MTB	100	100	37.5	50	25	3
SIT1688	MTB	100	75	50	50	37.5	2
SIT336	MTB	100	37.5	100	37.5	50	1.5
SIT149	MTB	50	50	25	50	50	0.5
SIT53	MTB	75	75	25	50	50	0.25
SIT54	MTB	75	50	25	50	37.5	3
SB1176	MB	50	50	50	50	25	0.09
SB1953	MB	25	12.5	37.5	12.5	12.5	1
SB0133	MB	37.5	75	25	12.5	50	2

Ob **=** *Ocimum basilicum***,** Ca = *Calpurnia aurea*, Cm = *Croton macrostachyus*, Aa = *Artemisia abyssinica*, Ec = *Eucalyptus camaldulensis*, RIF = Rifampicin, MTB = *M. tuberculosis,* MB = *M. bovis.*

### Minimum inhibitory concentration

The MIC of the extracts was determined for their antimycobacterial activity using resazurin as an indicator for *M. tuberculosis* and *M. bovis* strains viability in 96-well microplates. The MIC of 80% methanolic extracts of root of *C. aurea,* seeds of *O. basilicum,* leaves of *A. abyssinica, C. macrostachyus* and *E. camaldulensis* ranged from 25–100 μg/mL and 12.5-75 μg/mL, 25–100 μg/mL and 25–50 μg/mL, 6.25-50 μg/mL and 12.5-50 μg/mL, 12.5-100 μg/mL and 18.25-50 μg/mL and 6.25-50 μg/mL and 12.5-50 μg/mL, respectively for *M. tuberculosis* and *M. bovis* strains. The investigation showed that leaves of *E. camaldulensis* extract was the most active against both *M. tuberculosis* and *M. bovis* strains (MIC 6.25-50 μg/mL). The mean MIC results of 80% methanolic crude extracts of each plant showed significantly lower antimycobacterial activities in comparison to rifampicin for all strains (Table 2). Although all extracts were found less active than rifampicin, extracts of leaves of *A. abyssinica* and *E. camaldulensis* showed interesting antimycobacterial activity. However, reference strain of *M. tuberculosis H37Rv,* SIT73, SIT149, SIT149, SB1176 and SB1953 strains were comparatively the most susceptible strains to the extracts of the study plants with MIC ≤ 50 μg/mL. In contrast, SIT37 and SIT336 strains were the most resistant to extracts as compared to other strains. The comparative mean MIC values of plant extracts and rifampicin against clinical isolates and reference strain of *M. tuberculosis H37Rv* and *M. bovis* strains *is* shown in (Table 2).

Despite intense hard work to control TB disease, it remains one of the global health problems claiming 1.4 million human lives per annum. Furthermore, in the worldwide up to 50 million people are said to be infected with drug-resistant forms of TB with about 500,000 cases of multi-drug resistant MDR-TB per a year [5). Thus, with the emergence of the drug-resistant strains of MTC, lengthy of therapy, which is very hurtful to a patient’s health due to high levels of drug toxicity and its various adverse effects, the need to search for new effective anti-TB agents has become very necessary. Using medicinal plants for the treatment of TB offers a great hope to fulfill these needs because of their chemical diversity and they have been used for curing diseases for many centuries [11]. In addition, natural herbs continue to play a great significant role in the drug discovery and development of highly active antimycobacterial metabolites and they can be used as pure compounds or as crude materials [12].

In Ethiopia, many medicinal plants have been found which are used in the treatment of microbial infections including TB. It is our interest to report the anti-TB activity of these plants since these plants are reported from communities to treat TB and other respiratory infections. During ethnobotanical study these plants had been reported to possess antimicrobial activity. In the present study, the crude 80% methanol extracts of seeds of *O. basilicum,* root of *C. aurea*, and leaves of *C. macrostachyus*, *A. abyssinica* and *E. camaldulensis* showed promising antimycobacterial activity against *M. tuberculosis* and *M. bovis* strains. This may be due to the bioactive constituents, such as alkaloids, tannins, saponins, phenols, flavonoids and others that are present in the extracts [28,29]. Although the antibacterial activity of *O. basilicum, C. aurea*, *C. macrostachyus*, and *A. abyssinica* had been reported against other various pathogenic bacteria [30,31], no report was found during a literature search against mycobacterium strains apart from ethnobotanical reports on these plants. Therefore, this investigation would be the first report on their anti-mycobacterial activities. The crude 80% methanolic extracts of leaves of *E. camaldulensis* exhibited the most antimycobacterial activity against both *M. tuberculosis* and *M. bovis* strains. This could be due the bioactive tannins and saponins present in the extracts. This report agrees with the previous reports of hexane, chloroform, methanol extracts of *E. camaldulensis *[32,33]. In Ethiopia, the present work demonstrates antimycobacterial properties of these plants having therapeutic value in the treatment of TB. However, it is generally recognized that the more antimycobacterial activity of extracts depends on their lipophilic nature and showed better activity [20,34].

## Conclusions

Even though this investigation supports the traditional medicinal usage in the Ethiopian community and the medicinal uses of these plants in different regions of Ethiopia; to obtain the most promising antimycobacterial activity and to conclude their anti-TB activity, these plants should be fractionated with less polar solvents. In addition, further investigations should focus on isolating chemical constituents responsible for pharmacological activities and identifying the compounds eliciting the activity observed in these plants.

## Abbreviations

ALIPB: Aklilu Lemma Institute of Pathobiology; DMSO: Dimethyl sulphoxide; MDR-TB: Multi-drug resistant tuberculosis; MIC: Minimum inhibitory concentration; OADC: Oleic acid, Albumin, Dextrose, and Catalase; REMA: Resazurin microtiter assay; SIT: Spoligotype International Type; TB: Tuberculosis; WHO: World Health Organization; XDR-TB: Extensively drug resistant tuberculosis.

## Competing interests

The authors declare that they have no competing interests.

## Authors’ contributions

AG designed the study, participated in data collection, analysis and drafted the manuscript. MG, participated in study design, data collection, analysis and write-up. GA, participated in study design, data collection, data analysis, interpretation and write-up of the manuscript and critically revised the manuscript. AW, involved in laboratory work, data analysis and interpretation. All authors read and approved the final manuscript. AG is the guarantor of the paper.

## Pre-publication history

The pre-publication history for this paper can be accessed here:

http://www.biomedcentral.com/1472-6882/13/291/prepub
